# Elevated serum triglyceride and low-density lipoprotein cholesterol promotes the formation of colorectal polyps

**DOI:** 10.1186/s12876-019-1115-9

**Published:** 2019-11-21

**Authors:** Chenxi Xie, Pingwu Wen, Jingling Su, Qin Li, Yandan Ren, Yueyu Liu, Renze Shen, Jianlin Ren

**Affiliations:** 1 Department of Gastroenterology, Zhongshan Hospital Affiliated to Xiamen University, Xiamen, Fujian Province 361000 People’s Republic of China; 2 Department of Gastroenterology, Meizhou Affiliated Hospital of Sun Yat-sen University, Meizhou, Guangdong Province 514000 People’s Republic of China; 3 Guangzhou Center for Disease Control and Prevention, Guangzhou, Guangdong Province 510080 People’s Republic of China; 4 Renze Shen, Department of Stomatology, Zhongshan Hospital, Xiamen University, Xiamen, Fujian Province 361000 People’s Republic of China

**Keywords:** low-density lipoprotein cholesterol, triglyceride, colorectal polyp, advanced adenoma

## Abstract

**Background:**

Hyperlipidaemia may be a potential risk factor for the occurrence of intestinal polyps. This study aimed to evaluate correlation between lipidaemia and the formation of colorectal polyps.

**Methods:**

One hundred and fourteen patients with colorectal polyps and forty-eight healthy controls were included in this study. Colonoscopies were performed for all patients and controls within 1 week before blood samples were taken. The concentrations of serum lipids and lipoproteins were measured simultaneously using an automatic biochemical analyser. The colorectal lesions were classified based on pathological characteristics, and four types were identified in the study: hyperplastic polyp (HP), tubular adenoma (TA), tubulovillous adenoma (TVA) and adenoma with high-grade dysplasia (A-HGD). Advanced adenoma was classified according to the number, size and histological type of polyps.

**Results:**

The value of low-density lipoprotein cholesterol (LDL-C) was significantly higher in the group with advanced adenoma than in the controls (*p* < 0.05). Moreover, the LDL-C values in the HP and TA groups were higher when compared to that of controls (*p* < 0.05). Obesity, age, and increased TG and LDL-C were independent risk factors for the formation of colorectal polyps. The cut-off values of triglyceride (TG) and LDL-C to distinguish polyp patients from healthy controls were 0.96 mmol/L (AUC = 0.604, *p* = 0.036) and 3.05 mmol/L (AUC = 0.654, *p* = 0.002). The combined use of increased LDL-C and TG levels to distinguish polyp patients was effective, with a sensitivity of 50.0% and a specificity of 89.6% (AUC = 0.733, *p* < 0.01).

**Conclusions:**

Colorectal polyps are more often found in obese and older patients. Increased LDL-C and TG were correlated with the occurrence of polyps. Combination of the two serum indicators was useful to assess risk of colorectal lesions, maybe more effective in screening hyperplastic polyp, tubular adenoma and advanced adenoma.

## Background

Colorectal cancer (CRC) is the fourth cause of cancer death in the world [1], and CRC develops through the adenoma-carcinoma sequence. The risk of CRC increases in patients with large and multiple polyps, as well as in patients with villous, tubulovillous or high-grade dysplasia adenomas [1–3]. Although faecal immunochemical testing (FIT) screening can help reduce the mortality of CRC by more than 50%, there is no advantage in finding low-risk adenomas (LRAs) [4]. Colonoscopy has the ability to detect early-stage cancers and remove adenomas, but excessive surveillance can overwhelm social resources [5]. Nonetheless, knowledge of factors associated with polyp formation can help identify potential patients who need endoscopic screening.

Previous studies have shown that alterations in the gut microbiota can activate inflammatory signalling and increase the incidence of colorectal adenoma and metabolic syndrome (MetS) [6, 7]. Obesity and hyperinsulinaemia are the key elements of MetS. Other pathophysiological markers are dyslipidaemia and hypertension. Some studies have suggested that high body mass index (BMI) and hyperglycaemia may be independent risk factors for colorectal neoplasia [8–10]. However, these factors do not fully explain the variability in polyp risk, and the role of serum lipids is still not clear.

High levels of triglyceride and cholesterol, as well as low levels of high-density lipoprotein cholesterol (HDL-C), are potential markers for advanced adenoma, but this is controversial [10–13]. Low-density lipoprotein cholesterol (LDL-C) and apolipoprotein B (apoB) transport lipids to peripheral tissues, causing a cascade of chronic inflammation [14]. Higher levels of LDL-C and apoB may be associated with colorectal lesions, but this was not supported by the EPIC study [13, 15]. Thus, further study is needed to determine the role of dyslipidaemia in polyp occurrence.

Accordingly, the aims of the current study were as follows:

(i) to compare differences in serum levels of lipids and lipoproteins between patients and controls
(ii) to assess whether serum lipids and lipoproteins correlate with the incidence of advanced adenoma; and
(iii) to evaluate whether serum lipids and lipoproteins are associated with the formation of colorectal polyps.

## Methods

### Subjects

This was a prospective study. Consecutive outpatients who underwent colonoscopy from July 2015 to June 2017 were enrolled. One hundred and fourteen patients with colorectal polyps were included. Patients were excluded if they had the following conditions: taking lipid-lowering drugs; gastrointestinal tumours; previous abdominal surgery; and severe renal, cardiac or pulmonary disease. At the same time, another forty-eight volunteers with normal mucosa via colonoscopy and without digestive symptoms, cancer, severe systemic disorders or a history of gastrointestinal surgery were recruited as the control group.

The study protocol and the recruitment of the patients and controls were approved by the Ethics Committee of Zhongshan Hospital Affiliated to Xiamen University. Written informed consent was obtained from all individuals before starting any study procedure.

### Colonoscopy procedure

Colonoscopies were performed for all patients and controls within 1 week before blood samples were taken. All patients were required to consume a liquid diet for 24 h before the examination, and polyethylene glycol was used to complete the standard bowel preparation. The colonoscopy procedure was performed in our hospital by the same physician (Chenxi Xie). All lesions were removed by endoscopic resection, and biopsies were inspected under a microscope by experienced pathologists. Polyps were classified according to pathological characteristics. Four types of lesions were identified in the study: if the polyp is composed of proliferating regular glands under the microscope, it is classified as a hyperplastic polyp (HP); if the polyp is composed of an irregular small tubular structure, it is classified as a tubular adenoma (TA); if the polyp is composed of a more papillary structure with proliferative vascular connective tissue, it is classified as a tubulovillous adenoma (TVA); if the polyp includes malignant cells, but without evidence of interstitial infiltration, it is classified as a high-grade dysplasia (A-HGD).

If the patient had more than one histological type of lesion, the grouping was based on the neoplasia with the highest risk. Advanced adenoma (AA) was defined as the coexistence of more than two lesions or the presence of any colorectal polyps with a diameter of more than 10 mm or with high-risk pathology (tubulovillous or high-grade dysplasia changes) [1, 16].

### Assessment of metabolic syndrome markers

Blood lipids and lipoproteins including cholesterol (CHO), triglyceride (TG), high-density lipoprotein cholesterol (HDL-C), low-density lipoprotein cholesterol (LDL-C), apolipoprotein A1 (apoA1) and apolipoprotein B (apoB) were assessed in our study. Samples were taken under fasting conditions, and concentrations were measured simultaneously using a Hitachi Automatic Biochemical Analyzer 7000 with reagents and calibrators from Roche Diagnostics. All assays were performed according to the manufacturer’s protocols by an investigator blinded to the case-control status.

The status of smoking and alcohol intake were obtained based on participant self-reporting. Patients were considered to have a history of diabetes or hypertension if they recently used medicines to control related symptoms.

### Statistical analysis

Data were expressed as either the mean ± SD or median (interquartile range). One-way ANOVA was used to compare differences if the values for a metric followed a normal distribution; otherwise, the rank-sum test was used. Logistic regression analysis was employed to investigate the association of serum lipids and lipoproteins with the occurrence of colorectal polyps. A *p* value of less than 0.05 was considered statistically significant. The statistical analysis was accomplished using SPSS 20.0 (SPSS Inc., Chicago, IL, USA).

## Results

### Demographic characteristics of the patients

A total of one hundred and fifty-nine patients were screened by colonoscopy during physical examination. Twenty-seven patients with systemic disorders or a history of previous abdominal surgery and eighteen patients with digestive cancer were excluded. Forty-eight healthy controls with normal mucosa via colonoscopy were included in the analysis (Table 1).

**Table 1:** Baseline characteristics between controls and patients

	Without AA (39)	With AA (75)	Control (48)	*p* value
Age (years)	46.69 ± 10.40	51.81 ± 11.29*	44.81 ± 11.17	0.002
Male (%)	56.41%	76.39%*	50%	0.008
BMI	23.39*(22.43,25.26)	23.18*(20.96,25.15)	22.11 (19.14,23.17)	0.001

*p* < 0.05 means that the distribution of values in each group is not equal

An asterisk means that the difference is significant when compared to the controls separately

*AA* advanced adenoma

Age is expressed as the mean ± SD. BMI is expressed as the median (interquartile range)

### Comparison of serum levels of lipids and lipoproteins between patients with colorectal polyps and controls

Patients with polyps were older and had a higher BMI, with a higher proportion of males (*p* < 0.05). TG, LDL-C and apoB values were lower in the control group than in the patients (*p* < 0.05) (Table 2).

**Table 2:** Comparison of metrics between patients with polyps and controls

	patients (114)	Control (48)	*p* value
Age (years)	50.06 ± 11.21	44.81 ± 11.17	0.007
Male (n)	79	24	0.020
BMI (kg/m^**2**^)	23.38 (21.28,25.23)	22.11 (19.14,23.17)	0.000
With diabetes (n)	2	1	1.00
With hypertension (n)	13	1	0.105
Smoking (n)	24	7	0.339
Drinking (n)	18	3	0.099
CHO (mmol/L)	4.65 (4.10,5.24)	4.63 (4.16,5.31)	0.701
TG (mmol/L)	1.31 (0.99,1.83)	0.97 (0.75,1.74)	0.036
HDL-C (mmol/L)	1.25 (1.06,1.51)	1.37 (1.10,1.47)	0.244
LDL-C (mmol/L)	3.23 ± 0.82	2.81 ± 0.74	0.003
apoA (g/L)	1.37 ± 0.27	1.27 ± 0.33	0.063
apoB (g/L)	0.95 (0.82,1.10)	0.84 (0.77,0.96)	0.040

BMI, CHO, TG, HDL-C, and apoB are expressed as medians (interquartile range). Age, LDL-C and apoA are expressed as means ± SD

### Comparison of serum levels of lipids and lipoproteins between patients with or without advanced adenoma and controls

Values of CHO, HDL-C, apoA1 and apoB were similar between the patients and controls (*p* > 0.05). It seemed that TG was increased in the patient groups, but the differences were not significant when compared to that of controls(*p* > 0.05). The LDL-C value in the group with advanced adenoma was higher than that in the control group (*p* < 0.05), but the difference were not significant between patients with and without advanced adenoma (*p* > 0.05) (Table 3).

**Table 3:** Comparison of serum levels of lipids and lipoproteins between patients with or without advanced adenoma and controls

	Without AA (39)	With AA (75)	Control (48)	*p* value
CHO (mmol/L)	4.62 (3.85,5.12)	4.64 (4.15,5.37)	4.63 (4.16,5.31)	0.530
TG (mmol/L)	1.51 (1.04,1.92)	1.25 (0.96,1.70)	0.97 (0.75,1.74)	0.058
HDL-C (mmol/L)	1.18 (1.04,1.40)	1.26 (1.09,1.51)	1.37 (1.10,1.47)	0.287
LDL-C (mmol/L)	3.14 ± 0.83	3.28 ± 0.82*	2.81 ± 0.74	0.007
apoA (g/L)	1.39 ± 0.28	1.37 ± 0.26	1.27 ± 0.33	0.116
apoB (g/L)	0.92 (0.75,1.10)	0.95 (0.82,1.09)	0.84 (0.77,0.96)	0.112

*p* < 0.05 means that the distribution of values in each group is not equal

An asterisk means that the difference is significant when compared to the controls separately

*AA* advanced adenoma

CHO, TG, HDL-C, and apoB are expressed as medians (interquartile range). LDL-C and apoA are expressed as means ± SD

### Comparison of serum levels of lipids and lipoproteins between patients with different pathological types of lesions and controls

Differences in the values of CHO, TG, HDL-C, apoA1 and apoB between the patients and controls were not significant (*p* > 0.05). There was an increased trend of LDL-C values in all patient groups (*p* = 0.029), but the differences were not significant in the four polyp groups (*p* > 0.05). When compared to the controls separately, only the TA and HP groups with significant higher LDL-C values (*p <* 0.05) (Table 4).

**Table 4:** Comparison of serum levels of lipids and lipoproteins between patients with different colon lesions and controls

	TA (72)	HP (14)	TVA (17)	A-HGD (11)	Control (48)	*p* value
CHO (mmol/L)	4.64 (4.15,5.18)	4.85 (4.07,5.65)	4.12 (3.68,4.72)	4.77 (4.36,5.55)	4.63 (4.16,5.31)	0.349
TG (mmol/L)	1.41 (1.01,1.95)	1.10 (0.95,1.60)	1.31 (1.07,2.10)	1.16 (0.74,1.37)	0.97 (0.75,1.74)	0.081
HDL-C (mmol/L)	1.26 (1.06,1.52)	1.22 (1.07,1.38)	1.18 (0.84,1.49)	1.42 (1.04,1.90)	1.37 (1.10,1.47)	0.361
LDL-C (mmol/L)	3.26 ± 0.80*	3.32 ± 0.77*	3.00 ± 0.82	3.30 ± 1.09	2.81 ± 0.74	0.029
apoA (g/L)	1.39 ± 0.26	1.36 ± 0.23	1.28 ± 0.29	1.42 ± 0.34	1.27 ± 0.33	0.173
apoB (g/L)	0.97 (0.82,1.13)	0.97 (0.81,1.10)	0.88 (0.73,0.96)	1.00 (0.67,1.20)	0.84 (0.77,0.96)	0.149

*p* < 0.05 means that the distribution of values in each group is not equal

An asterisk means that the difference is significant when compared to the controls separately

*HP* hyperplastic polyp, *TA* tubular adenoma, *TVA* tubulovillous adenoma, *A-HGD* adenoma with high-grade dysplasia

CHO, TG, HDL-C, and apoB are expressed as medians (interquartile range). LDL-C and apoA are expressed as means ± SD

### Association of serum levels of lipids and lipoproteins with the occurrence of colorectal polyps

The cut-off value of TG for distinguishing the patients from controls was 0.96 mmol/L (AUC = 0.604, *p* = 0.036). The sensitivity was 79.8%, and the specificity was 50.0%.

The cut-off value of LDL-C for distinguishing the patients from controls was 3.05 mmol/L (AUC = 0.654, *p* = 0.002). The sensitivity was 56.1%, and the specificity was 75%.

The cut-off value of apoB for distinguishing the patients from controls was 0.98 mmol/L (AUC = 0.602, *p* = 0.040). The sensitivity was 44.7%, and the specificity was 79.2%.

Logistic regression analysis was applied to identify potential risk factors for colorectal lesions. The three cut-off values above were used to divide all the participants into two groups separately. BMI > 25 kg/m^2^ was used as the demarcation of obesity, and the participants were divided into four groups according to age (≤40 years; 41–50 years; 51–60 years; > 60 years).

The results showed that obesity, age, and increased TG and LDL-C were independent risk factors for the occurrence of colorectal polyps (for obesity, OR = 3.55, 95%CI 1.12–11.24; for age categories, OR = 1.58, 95%CI 1.09–2.30; for TG > 0.96 mmol/L OR = 2.64, 95%CI 1.20–5.84, and LDL-C > 3.05 mmol/L, OR = 2.89, 95% CI 1.29–6.39. all *p* < 0.05).

In addition, the combined use of increased LDL-C and TG to distinguish patients with polyps was effective, with a sensitivity of 50.0% and a specificity of 89.6% (AUC = 0.733, *p* < 0.01).

## Discussion

Transformation of adenoma to CRC is a long process. Endoscopic resection of precancerous lesions is beneficial for reducing the incidence of CRC, but determining suitable patients is still difficult. Moreover, the correlation between lipidaemia and colorectal neoplasia is controversial [10, 17]. In this study, we evaluated potential factors correlated with the formation of colorectal polyps and found that higher serum levels of LDL-C and TG favoured neoplasia.

During inflammation, changes occur in lipids, not only in quantity but also in quality. Chronic inflammation impairs the normal transport of cholesterol and stimulates compensatory changes, such as the synthesis of LDL-C and VLDL-C (very low-density lipoprotein cholesterol), resulting in the accumulation of TG in intestinal cells [14], which causes harmful reactions. Dyslipidaemia can induce the production of inflammatory cytokines, such as interleukin-6 and tumour necrosis factor-α, and reduce the anti- inflammatory cytokines, such as Interleukin-10 [14, 18]. These changes create a cellular environment conducive to neoplasia. So it was not surprising that the serum levels of LDL-C and TG were higher in patients with polyps.

Previous studies have shown a positive association between oxidative stress and LDL-C [19, 20], and LDL-C may be a cause of increased intracellular oxidative stress, whereby reactive oxygen species (ROS) induce lipid peroxidation and activate transcription factors [21]. This may facilitate cell proliferation and evasion from apoptosis. Therefore, increasing LDL-C can be observed in early stages of colorectal carcinogenesis, such as hyperplastic polyp and tubular adenoma. The intermediates produced by ROS attacking lipids can couple to DNA and induce genotoxic lesions, which promote carcinogenesis through the modulation of gene expression and mutation [22]. LDL-C is beneficial to oxidative damage, so it was reasonable to find the value increasing in patients with advanced adenoma.

It seemed that TG was increased in patients with advanced adenoma, but the difference was not significant when compared to that of controls. In fact, previous researches on the correlation between TG and colorectal lesions are not consistent [15, 23, 24]. In the Metabolic Syndrome and Cancer Project, TG was related to the increased risk of neoplasia [23]. But Chung YW et al. found that there was a negative association between TG levels and the risk of cancer [24], which may be due to malnutrition and metabolic changes in patients with advanced lesions. In the EPIC study, the results may have been affected by the postprandial variability of TG because the samples were taken without fasting [15]. Because TG is susceptible to various factors, it should be combined with other indicators in monitoring polyp occurrence.

Liu CS et al. reported that dyslipidaemia is an independent risk factor for colorectal adenoma [25], consistent with our results. In our study, the sensitivity of TG was high, but the specificity was poor; the reverse was observed for LDL-C. Nonetheless, the AUC was larger when the two indicators were combined used to distinguish polyp patients compared to either alone. This combination with very high specificity may provide useful information for surveillance and may help exclude patients for whom there is no urgency for colonoscopy screening. In this situation, FIT may be the preferred method of examination.

Other potential risk factors associated with the formation of polyps were also analysed. Increased body weight is now recognized as a factor correlated to the development of CRC, and it plays an important role at the pre-cancer stage [8, 26]. This was consistent with our results. Chronic hyperinsulinaemia associated with obesity increases the bio-active levels of IGF-1 (insulin-like growth factor 1), which can promote tumour growth [27]. The incidence of colorectal adenoma increases with age according to the European and Asia-Pacific guidelines [28, 29], and this was supported by the positive correlation between higher age and the formation of polyps in our study. However, differences in adenoma prevalence between the sexes are still controversial. Although a few studies have suggested that males are at a higher risk of developing colorectal adenoma [29–31], we did not find a significant association between male sex and polyp occurrence.

Although it appeared that there were more people with unhealthy lifestyles in the colorectal lesion group, the differences were not significant. Alcohol consumption may not be an independent risk factor for adenoma occurrence [32]. Many prediction models for colorectal neoplasia include smoking as a risk factor, but their discriminatory power is weak [32]. Smoking may promote polyp proliferation, but likely only if the habit has lasted decades [28]. In fact, a better risk prediction model to inform adenoma screening strategies remains to be determined [32].

Our study has some limitations. First, age and body weight between the patients and controls were not comparable, which may have impacted the reliability of our conclusion. Therefore, we used logistic regression analysis to exclude the influence of confounding factors and found that hyperlipidaemia was correlated with adenoma formation. Second, this was a single-centre study, and the sample size was relatively small. We described the observed results but lacked follow-up data for patients after polypectomy. Comparison of the recurrence rates between patients with or without hyperlipidaemia would be useful in supporting increased LDL-C and TG levels as potential markers for colorectal adenoma screening.

## Conclusions

In summary, our study suggests that the incidence of colorectal polyps increases with obesity and age. Increased LDL-C and TG correlated with polyp occurrence. LDL-C was less sensitive than TG in distinguishing patients from healthy controls but showed higher specificity. Hyperlipidaemia was a risk factor of colorectal lesions, especially for hyperplastic polyp, tubular adenoma and advanced adenoma.

## Data Availability

The datasets used and/or analysed during the current study are available from the corresponding author on reasonable request.

## Abbreviations

AA: advanced adenoma
A-HGD: adenoma with high-grade dysplasia
apoA1: apolipoprotein A1
apoB: apolipoprotein B
BMI: body mass index
CHO: cholesterol
CRC: colorectal cancer
FIT: faecal immunochemical testing
HDL-C: high-density lipoprotein cholesterol
HP: hyperplastic polyp
IGF-1: insulin-like growth factor 1
LDL-C: low-density lipoprotein cholesterol
LRA: low-risk adenomas
MetS: metabolic syndrome
ROS: reactive oxygen species
TA: tubular adenoma
TG: triglyceride
TVA: tubulovillous adenoma
VLDL-C: very low-density lipoprotein cholesterol
